# Determinants of Computed Tomography Head Scan Ordering for Patients with Low-Risk Headache in the Emergency Department

**DOI:** 10.7759/cureus.1760

**Published:** 2017-10-09

**Authors:** Meaghan J Mackenzie, Rashi Hiranandani, Dongmei Wang, Tak Fung, Eddy Lang

**Affiliations:** 1 Medicine, University of Calgary; 2 Medicine, University of Ottawa; 3 Alberta Health Services, University of Calgary; 4 Information Technology, University of Calgary; 5 Emergency Medicine, University of Calgary

**Keywords:** choosing wisely, headache, emergency medicine, neuroimaging, head ct

## Abstract

Background

Many specialty societies have found that neuroimaging in headache is a low-value intervention for benign presentations. This study describes factors that influence Emergency Room (ER) physicians’ adherence to Choosing Wisely (CW) recommendations for low-risk headache patients presenting to Calgary’s Emergency Departments (EDs). Emergency medicine has yet to address imaging in headache as a CW topic; however, this study may inform that decision.

Methods

Data were retrospectively collected for all patients presenting to Calgary EDs with headaches from April 1, 2014 to March 31, 2016. Patients were deemed low-risk by virtue of discharge home from the ED, age < 50, and no lumbar puncture (LP), trauma, neurology, or neurosurgery consult or red flags on history. The primary outcome was computed tomography (CT) ordering rates with an eye to medical doctor (MD) practice variation. Patient, physician, and environmental factors were analyzed to compare patients who did and did not receive a CT.

Results

Two thousand seven hundred and thirty-four headache patients met the eligibility criteria. A total of 117 Calgary ER physicians were included, all of whom had seen 10 or more headache patients over the study period. Physician practice variation was vast, with a mean ordering rate of 38.0% and a range of 0% to 95% (M = 39.0%, IQR = 21.0%). CTs were ordered more often in males than females (39.9%; 34.1%; p = 0.002) and in patients presenting during the day and evening (38.1%; 39.0%) compared to the night (29.7%; p < 0.001). Patients were divided into quartiles by age, with the oldest group (41.6 - 50 years) receiving significantly more head CTs (45.1%) than the other quartiles (34.9%; 34.9%; 27.5%; p < 0.001). Longer triage-to-discharge times were associated with an increase in CT ordering rates (12% for < 2.95 hours; 35% for > 4 hour wait; p < 0.001). Lastly, patients who did not have a CT were more likely to revisit the ED within seven days compared to those who did (6.9% vs 4.0%; p = 0.003), but their seven-day admission rate was unaffected (0.6% in the group that got CTs and 0.3% in the group that did not get a CT). Time to assessment, the day of the week, physician gender, years of experience, and training program did not influence CT ordering practices.

Conclusion

To our knowledge, this is the first study to assess how patient, physician, and environmental factors relate to the use of CT scans in low-risk headaches presenting to the ED. CW guidelines are not optimally adhered to, and the findings in this study findings may inspire new ideas for maximizing the judicious use of healthcare resources.

## Introduction

Increasingly, evidence-based guidelines and algorithms are being implemented in Canadian Emergency Departments to encourage a resource-conscious approach to diagnostic imaging [1-3]. These decision-making tools have been designed through the collaboration of several specialist societies in order to ensure that patients are safely and appropriately evaluated. Choosing Wisely Canada (CWC) emphasizes the importance of selecting the right type of intervention, if any, for the particular clinical scenario. The aim of these recommendations is to ensure the delivery of high-quality healthcare by increasing dialogue between doctors and their patients with regard to the concept that more may not always be better in healthcare [3]. In 2014, the radiology sub-group within the CWC campaign released recommendations—not specific to Emergency Department (ED) patients—regarding the need for imaging tests in patients with headaches. They suggest imaging be performed in the following situations: headaches associated with focal neurological findings; a worrisome headache that cannot be diagnosed based on physical exam and history; a severe headache where one feels a bursting sensation inside the head; a new onset headache, unlike previous headaches in patients over the age of 50; a headache that comes on after physical activity; or a headache that is associated with loss of control, a seizure, or change in speech or alertness [3].

Despite the capabilities of imaging modalities in diagnosing serious pathology related to headaches, it is widely accepted that the most clinically important diagnostic tools in evaluating benign headaches are a detailed history of the patient’s symptoms and a neurological exam [4]. If properly implemented, the evidence-based recommendations set out by CWC could optimize diagnostic imaging use, thereby decreasing unnecessary costs to our healthcare system, limiting unnecessary radiation exposure to the patient, and ultimately resulting in better patient care.

The lifetime prevalence of headaches is estimated at 66%, and roughly 2.6 million Canadian women, and 1 million men, experience migraine headaches [5]. While relatively common, headaches can also be an indication of a life-threatening intracranial pathology [6], and therefore it is critical to appropriately diagnose, manage, and treat this condition. The aim of obtaining neuroimaging studies for headache patients is to identify treatable pathology, such as tumours, vascular malformations, aneurysms, subarachnoid haemorrhage, cerebral venous sinus thrombosis, subdural and epidural hematomas, infections, stroke, and hydrocephalus [7]. Fear of missing these low-probability, yet life-threatening, diagnoses is one of the most commonly self-identified reasons for unnecessary diagnostic imaging procedures carried out in the ED and, therefore, are a reason to expect suboptimal rates of adherence to imaging guidelines [8].

The purpose of our study was three-fold. First, we set out to discover and describe a low-risk cohort of patients identifiable via administrative data available through Calgary Emergency Departments. We wanted to describe the patient, physician, and environmental factors that influence the described adherence rates to CW head computed tomography (CT) ordering recommendations. Lastly, emergency medicine has yet to choose imaging in headache as a topic to collaborate on; this study may inform that decision.

## Materials and methods

Data were retrospectively collected on all patients that presented to any of the four Calgary Emergency Departments (Foothills Medical Centre, Rockyview General Hospital, Peter Lougheed Centre and South Health Campus) with low-risk headaches from April 1, 2014 to March 31, 2016. To provide some context, during this time period, there were 10,063 visits for headache (both low- and high-risk) to Calgary Emergency Departments, with a total of 192 admissions. Of this group, 2,734 were determined to be low-risk headache patients. It should be noted that the Calgary EDs included have 24/7 access to ordering in-hospital CT scans that are read by radiologists.

Administrative data were collected via Sunrise Clinical Manager (SCM) and Sunrise Emergency Care (SEC), electronic order systems used in all Calgary EDs in order to track patient information, department flow, and order entry data. We used the tenth edition of the International Classification of Disease (ICD10) to select our patient cohort. Patients presenting with a complaint of headache were included, along with those with a discharge diagnosis of headache or migraine. We modified the cohort to select only low-risk patients, by applying comprehensive inclusion and exclusion criteria, listed in Table 1. There were 2,734 headache patients that met the eligibility criteria, as well as 117 physicians, all of whom had seen a minimum of 10 headache patients over the study period. Patient factors (age, gender, and pain rating of headache) were analyzed along with specific physician factors (gender, years of practice, and type of residency program: Fellow of the Royal College of Physicians of Canada (FRCPC) versus Canadian College of Family Physicians – Emergency Medicine certificate (CCFP-EM)), and environmental factors (day of the week, time of day, presenting hospital, length of stay, and seven-day revisit and admission rate) in order to compare patients that received CT scans for low-risk headaches to those who did not. This was performed using Statistical Package for the Social Sciences (SPSS; IBM, New York, USA) for chi-squared and t-test analyses.

**Table 1:** Patient inclusion and exclusion criteria CTAS Score (Canadian Triage and Acuity Scale): A five-level scale used to assign valid acuity scales to patients in the department. One is the most acute, and five is the least. ED: Emergency Department; INR: international normalized ratio; PTT: prothrombin time; CSF: cerebral spinal fluid

Inclusion Criteria	Exclusion Criteria
Patients > 18 years	INR > 1.2, PTT > 40 seconds
Patients < 50 years	Post lumbar puncture headache
Discharge home from ED	Pregnant patients
Atraumatic headache	CTAS of 1
CTAS 2-5	Neurology/neurosurgery consult
	Orders for CSF analysis or results from CSF analysis
	Hospital admission
	Active or previous cancer (including benign brain tumors and pseudotumor cerebri)
	-Charts that mention*:
	- patient c/o of "bursting sensation" with their headache
	- note of "other serious symptoms”, such as a loss of control, a seizure or fit, or a change in speech or alertness"
	- headaches that come on after physical activity
*In accordance with CWC guidelines	

Researchers accessed a limited data set, with all individual patient identifiers previously removed. A project ethics community consensus initiative (ARECCI) is an ethics screening tool that was accessed prior to initiating the project [9]. With an ARECCI score of two, our project is considered to pose a minimal ethical risk. Accordingly, the recommended action was to appropriately manage patient information within the confines of our project, to minimize patient risk. We did so by erasing all identifiable patient information from the data we analyzed, and by keeping all patient information password-protected and limited to the authors of this paper.

## Results

Of the 2,734 patients included in the study, 988 of them received CT head imaging, making the overall ordering rate 36% in this low-risk cohort.

Among the 2,734 headache patients that were included in the study, 969 (35.4%) were male and 1,765 (64.6%) were female. This group’s overall mean age was 34 years (SD = 8.7). The mean age of the group who underwent CT was 35 years of age (SD = 8.69). The mean age of the group that did not receive a CT head was 33 years of age (SD = 8.68). Of the 117 Calgary emergency medicine (EM) physicians that were included in the study, 95 (81.2%) were males and 22 (18.8%) were females. The mean age of this group was 42.9 (SD = 9.8) (range 29 - 66). The mean number of years of practice for this physician group was 9.14 (SD = 9.1) (Table 2).

**Table 2:** Physician and patient characteristics CCFP-EM: Canadian College of Family Physicians – Emergency Medicine certificate, FRCPC: Fellow of the Royal College of Physicians of Canada

	No. (%)
Physician Characteristics	
Gender	
Male	95 (81)
Female	22 (18)
Mean age	42.9
Mean number of years of practice	9.1
Program of training	
CCFP-EM	73 (63)
FRCPC	44 (37)
Patient Characteristics	
Gender	
Male	969 (35)
Female	1765 (65)
Age	34.1

**Figure 1:**
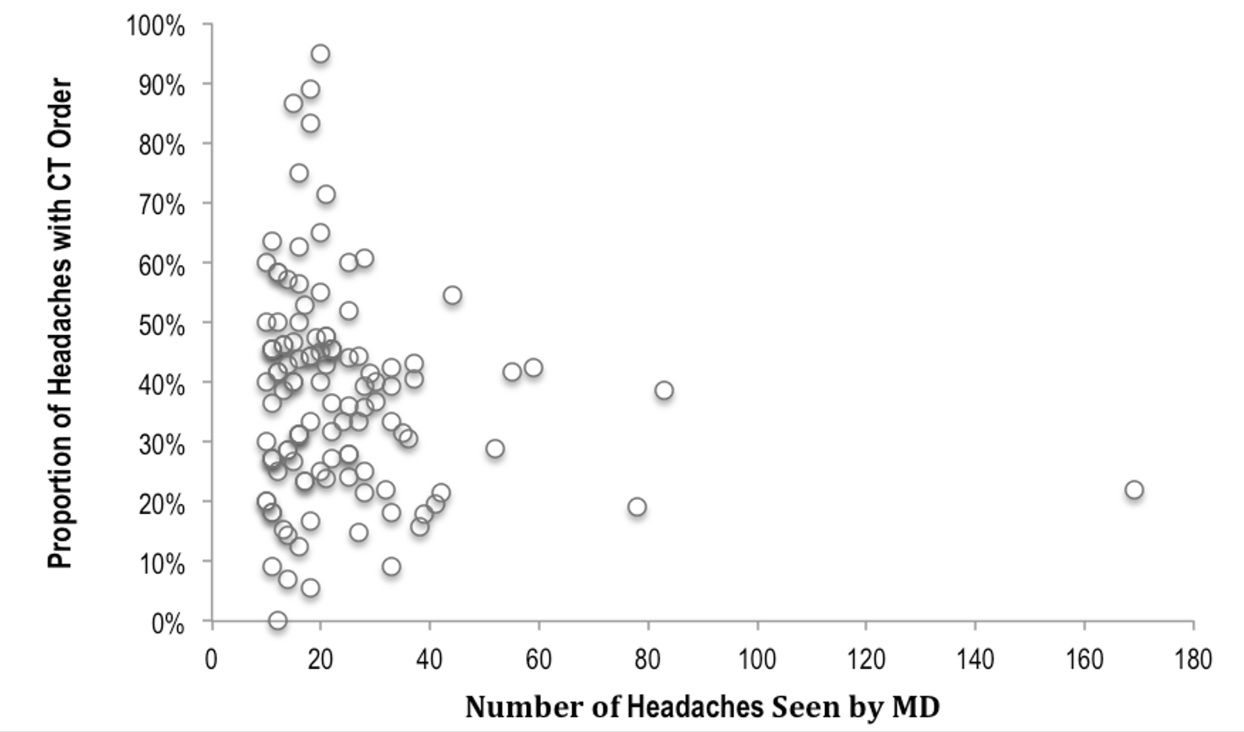
Proportion of head CTs ordered by number of headaches seen by each MD. Mean ordering rate was 38.0%, range 0-95% (M = 39.0%, IQR = 21.0%) CT: computed tomography MD: medical doctor

There was no significant difference in average head CT ordering rates related to gender, years of practice, or type of training program of the 117 physicians included in the study (Table 3). The physician mean ordering rate was 38.0% with a range of 0% to 95% (M = 39.0%, IQR = 21.0%) (Figure 1).

**Table 3:** Comparison of patient, environmental, and physician factors between patient groups that did and did not receive CT scans of the head CT: computerized tomography

	CT	No CT	Total		
	No. (%)	No. (%)		X2(df)	p
Patient factors					
Patient gender				9.39(1)	0.002*
Male	387 (39.9)	582 (60.1)	969		
Female	601 (34.1)	1164 (65.9)	1765		
Total	988	1746	2734		
Patient age				87.07(3)	<0.001*
≤ 27.22	203 (29.7)	481 (70.3)	684		
27.23-33.74	237 (34.9)	443 (65.1)	680		
33.75-41.62	240 (34.9)	447 (65.1)	687		
≥41.63	308 (45.1)	375 (54.9)	683		
Total	988	1746	2734		
Pain score				19.17(2)	<0.001*
Mild (1-3/10)	56 (44.1)	71 (55.9)	127		
Moderate (4-6/10)	199 (42.0)	275 (58.0)	474		
Severe (7-10/10)	439 (32.2)	923 (67.8)	1362		
Total	694	1269	1963		
Environmental factors					
Day of week				0.057(1)	0.812
Weekday	720 (36.3)	1265 (63.7)	1985		
Weekend	268 (35.8)	481 (64.2)	749		
Total	988	1746	2734		
Time of day				19.37(2)	<0.001*
Day (Hours)	317 (38.1)	514 (61.9)	831		
Evening (Hours)	445 (39.0)	696 (61.0)	1141		
Night (Hours)	226 (29.7)	536 (70.3)	762		
Total	988	1746	2734		
Presenting site				5.99(3)	0.112
Foothills Medical Centre	300 (39.1)	468 (60.9)	768		
Peter Lougheed Centre	236 (33.7)	464 (66.3)	700		
Rockyview General Hospital	242 (37.2)	409 (62.8)	651		
South Health Campus	210 (34.1)	405 (65.9)	615		
Total	988	1746	2734		
Revisit rates in next 7 days				9.13(1)	0.003*
No	948 (96.0)	1626 (93.1)	2574		
Yes	40 (4.0)	120 (6.9)	160		
Total	988	1746	2734		
Admission rates in next 7 days				1.00(1)	0.316
No	982 (99.4)	1740 (99.7)	2,722		
Yes	6 (0.60)	6 (0.30)	12		
Total	988	1746	2,734		
Triage to discharge time				179.11(3)	<0.001*
≤2.95	119 (12.0)	558 (32.0)	677		
2.96 - 4.18	230 (23.3)	461 (26.4)	691		
4.19 - 5.56	293 (29.7)	389 (22.3)	682		
>5.56	346 (35.0)	338 (19.4)	684		
Total	988	1746	2734		
Triage to MD assessment time				6.16(3)	0.104
≤1.02	264 (26.7)	413 (23.7)	677		
1.02 - 1.80	261 (26.4)	432 (24.7)	693		
1.80 - 2.87	236 (23.9)	447 (25.6)	683		
>2.87	227 (22.9)	454 (26.0)	681		
Total	988	1746	2734		
Physician factors					
Gender of assessing physician				0.311(1)	0.577
Male	844 (35.9)	1505 (64.1)	2349		
Female	144 (37.4)	241 (62.6)	385		
Total	988	1746	2734		
Training program of assessing physician				0.14(1)	0.707
Canadian College of Family Physicians -Emergency Medicine Certificate	646 (35.9)	1154 (64.1)	1800		
Fellow of the Royal College of Physicians of Canada	342 (36.6)	592 (63.4)	934		
Total	988	1746	2734		
Physician years of practice				1.18(1)	0.278
≤6.5	543 (35.3)	997 (64.7)	1540		
>6.5	445 (37.3)	749 (62.7)	1194		
Total	988	1746	2734		

Patients were divided into quartiles by age, with the oldest group receiving significantly more CT head scans (45.1%) than the other quartiles (34.9%; 34.9%; 29.7%; p < 0.001). Ordering rates were also influenced by patient gender, with more CTs ordered for males relative to female patients (39.9%; 34.1%; p = 0.002). Lastly, CTs were ordered more often for patients who reported mild/moderate amounts of pain (44.1%; 42.0%), than those in reportedly severe pain (32.2%; p < 0.001). (Table 3).

CTs were ordered more often in patients presenting during the day (07:00 - 14:59) and evening (15:00 - 22:59) (38.1%; 39.0%) versus at night (23:00 - 06:59) (29.7%; p < 0.001). Longer length of stay (from triage time to that of discharge) was associated with significantly more CT scans (Table 3) (proportion of CTs: 0 - 2.95 hrs = 12%, 2.96 - 4.18 hrs = 23.3%, 4.19-5.56 hrs = 29.7%, > 4hrs = 35%; p < 0.001). However, assessment times (from triage to MD assessment) were not associated with a change in CT ordering rates (Table 3). Although patients were more likely to revisit the hospital within seven days of the first presentation if they did not get an initial CT (6.9% vs 4.0%, p = 0.003), there was no effect on their likelihood of being admitted to hospital. Of the 2,634 patients included in the study, 160 revisited, and 120 of those patients did not receive an initial CT scan. CT head scans ordering practices were unaffected by site or the day of the week that the patient presented (Table 3).

## Discussion

The results show that several factors appear to affect emergency physicians' judgment when deciding whether or not to order a CT scan of the head in patients presenting with low-risk headaches.

From a quality improvement perspective, the most impressive of our results was the substantial practice variation amongst the physicians included in the study. The range for ordering practices is vast (from 0% - 95%), showing significant inconsistency in diagnostic approaches to benign headaches. This highlights the possibility that a headache patient’s chances of receiving a head CT scan may have more to do with which physician is working at the time that they present, than the symptoms they are presenting with. As mentioned above, this wide practice variation may be based on differences in physicians’ threshold for missing headaches due to life-threatening intracranial pathology. We originally hypothesized that physician experience—represented by years of practice or program of training—might explain part of the variation; however, this was not the case. Instead, physicians’ adherence to practice guidelines is multifactorial. Factors may include lack of physician knowledge; clinical scenarios not covered by guidelines; and some physician’s experiential disagreement with the recommendations [10-11]. However, Rosenburg, et al. [12] have shown that there has been a decrease (from 14.9% to 13.4%) in imaging in uncomplicated headache in the United States since the publication of the related CW recommendations. This modest decrease demonstrates that publishing guidelines can improve responsible use of resources, but it is widely recognized that measures greater than publication alone are needed in order to make further improvements [1-14]. It has been suggested that the most change can be made via guidelines by choosing interventions or procedures that have high baseline rates of inappropriate use [15]. With an ordering rate of 36% in Calgary, imaging in uncomplicated headaches is a topic that warrants more attention. Therefore, if EM were to choose imaging in headache as a CW topic to collaborate on, perhaps this would increase the awareness as well as the applicability of the recommendations for practicing EM physicians. 

With regard to patient factors, there was a significant increase in head CT scan ordering rates in relation to increasing patient age. This finding is not surprising, as the risk of sinister intracranial processes causing headache increases with age. Males received significantly more CT heads than females with similar presentations of low-risk headaches. In general, females experience more primary headaches (e.g., migraines, tension headaches, cluster headaches) than do males [5] and present more often to the Emergency Department because of this condition. We hypothesize that this gender difference may be the result of detection bias; because females are more likely to present with primary headaches, physicians are more likely to accept a non-sinister mechanism and are more comfortable not ordering a CT scan of the head—with the opposite being true in males.

Of the 2,734 patients included in the study, we had pain scores (scale from 1 to 10) for 1,963 of them. Patients who reported mild or moderate amounts of pain (1 - 3/10; 4 - 6/10) received significantly more CT scans than did those who reported being in severe pain (7-10/10). Additionally, the average pain score for patients that received CT scans was 7/10, whereas the average score for the patients that did not receive scans was 7.42/10. The results related to pain scoring are difficult to interpret, as we were not able to get a pain score from all patients. It would be reasonable to assume that the missing data is more likely to come from low acuity pain patients, as they are in little to no visible distress and the interviewer can easily infer this, so a pain scale rating was likely not elicited. This omission may have skewed the average pain scores higher in the group that did not receive scans. If pain scores were recorded on all patients, we would likely see a more predictable pain pattern: higher head CT scan ordering rates in patients with higher pain scores. However, with the missing data, we were not able to make conclusions either way.

In terms of environmental factors, it was noted that significantly more head CT scans were ordered during the day and evening, than during the night, even when controlling for differences in frequency of visits throughout the day. In Calgary, radiologists read CT scans throughout the night; therefore, this result cannot be explained by a lack of resources. It was found that with increasing assessment times, there was no significant increase in ordering rates. However, with increasing discharge times, there was a significant increase in the proportion of CT scans ordered. This may simply be attributed to delays to discharge secondary to the time required for increased investigation (i.e., CT scan). 

We found that patients with low-risk headaches who did not receive a CT scan were significantly more likely to revisit the ED within seven days. Though the total number of patients revisiting was relatively small (160 total; 120 of those did not receive a CT for their initial visit), we estimate that the cost of 120 patients revisiting is likely less financially taxing on the system than scanning all patients presenting with a low-risk headache. Admission rates of those same patients within seven days of the first presentation was unaffected by head CT scan ordering rates. Day of the week or hospital site did not affect ordering practices.

Taken together, it is well recognized that there are patient, systemic, and physician factors that contribute to inappropriate head CT ordering in low-risk headache patients. In an era of increasing access to information, healthcare professionals are working with a new group of patients. Patients are increasingly informed about the pathology of disease and the interventions available in the ED to rule out such pathology. This access to information can create an expectation in some patients that everything possible must be done in order to rule out any and all sinister causes of their headache, no matter how clinically unlikely such a scenario may be. 

Physicians have the added responsibility of balancing their clinical decision-making with the medical and legal ramification of missing something life-threatening by not ordering neuroimaging tests. Managing flow in the ED is another expectation of emergency physicians, and ordering a CT head can sometimes be quicker than watching and waiting by re-examining a patient throughout the shift. Lastly, systematic factors play a major role in these ordering practices: for instance, expectations from consulting services that imaging be done prior seeing a patient; prolonged wait times; and sensationalized media reports about the standard of care of a department. In this case, it is not just physicians, but ED managers that have to balance public expectations with coordinating smoothly run departments.

The results of our study highlight important implications for ED managers and physicians. SCM (the Calgary zone electronic medical record) already has prompt questions (related to the Canadian CT head rule) when ordering a scan on a patient to force physicians to think twice about their order (i.e., Glasgow Coma Score < 15, two hours after injury, suspected basal/open/depressed skull fracture, etc). It would be valuable to include specific questions around red flags for low-risk headache as well (i.e., thunderclap headache, history of cancer, etc.). Additionally, this could go a step further by creating these same sorts of prompts for patients. For example, advertising around the department on the red flags related to headaches, as well as the adverse effects of invasive investigations, could force the patient to address the risks and benefits of the intervention. As headaches are such a common presentation to the ED, investment in increasing patient and physician awareness of unnecessary testing is a critical first step to mitigating this rapidly evolving this problem.

This study raises several important questions regarding CT head ordering practices in Calgary ED departments related to the CWC recommendations. However, some key limitations to the study should be noted. This study was not a formal chart review, limiting the amount of patient information that was considered in the study. This also means that our exclusion of patients was subject to the charting inconsistency of physician descriptions of patient presentations. Furthermore, as this was a retrospective study, the low-risk cohort was determined after—and not before—all the patient visits. Similarly, the statistical analysis was conducted retrospectively. As this study was confined to Calgary, it may lack applicability to other centers, with different populations, training programs, or access to healthcare resources.

## Conclusions

Choosing Wisely Canada is an initiative to reduce the overuse of unnecessary or harmful patient interventions. However, we know that the guidelines set out by CWC are not always optimally adhered to. This study draws attention to vast practice variations regarding the approach to benign headaches in terms of imaging and, therefore, creates a target for quality improvement via ED-specific CW guidelines on this topic. It is clear that adherence to CW recommendations is suboptimal with multiple culprits, including patient, physician, and systematic factors. In order to continue to deliver high-quality—as well as sustainable—healthcare, CT head ordering practices need to be optimized.
